# Characterization of Newly Developed Zinc Composite with the Content of 8 wt.% of Hydroxyapatite Particles Processed by Extrusion

**DOI:** 10.3390/ma13071716

**Published:** 2020-04-06

**Authors:** Jan Pinc, Jaroslav Čapek, Vojtěch Hybášek, Filip Průša, Klára Hosová, Jan Maňák, Dalibor Vojtěch

**Affiliations:** 1 Department of Metals and Corrosion Engineering, University of Chemistry and Technology, 166 28 Prague, Czech Republic; hybasekv@vscht.cz (V.H.); Prusaf@vscht.cz (F.P.); hosovak@vscht.cz (K.H.); vojtechd@vscht.cz (D.V.); 2 FZU–Institute of Physics of the Czech Academy of Sciences, 182 21 Prague, Czech Republic; capekj@fzu.cz (J.Č.); manak@fzu.cz (J.M.)

**Keywords:** zinc, metal-matrix composite, biodegradable metals

## Abstract

Zinc and its alloys belong to a group of biodegradable materials, which can be potentially used for the preparation of temporary orthopedic implants. The research of biodegradable zinc materials revealed a lot of limitations; however, the new processing approaches of those materials can enhance their properties, which are insufficient for now. In this study, the zinc composite with 8 wt.% of hydroxyapatite (Zn/HA8) prepared for the first time by extrusion process was characterized from the point of view of the structural, mechanical and corrosion properties. The extrusion process led to good integrity of the interfaces between the zinc and hydroxyapatite particles. Mechanical behavior confirmed the role of hydroxyapatite as a defect in the material structure, which led to a decrease of the Zn/HA8 mechanical properties by approximately 30% (compressive yield strength (CYS) = 154 MPa Zn, 113 MPa Zn/HA8). Despite that, the Zn/HA8 composite showed sufficient mechanical properties for cancellous bone replacement and reached the lower limit for cortical bone. Additionally, the presence of hydroxyapatite caused the preferential precipitation of hydroxyapatite (HA) from the solution and can lead to a significant enhancement of the tissue/implant interface interactions.

## 1. Introduction

Zinc and its alloys show remarkable potential to become a new generation of biodegradable materials [1,2,3,4]. The advantages of those materials, such as an almost suitable corrosion rate or nonparticipation of hydrogen in the corrosion process under the physiological conditions, are well known [5,6]. Despite the suitable corrosion properties, the application of as-cast zinc could be limited due to insufficient mechanical performance for the applications in the implantology [7]. Some in-vitro studies also suggest that pure zinc can exhibit insufficient biocompatibility [8,9]. Therefore, those limitations have to be solved and sufficient mechanical, corrosion and biological properties of Zn-based materials have to be achieved before implantation.

It is well known that a high concentration of zinc (approximately over 80 µM) can negatively affect the viability and adhesion of cells to the material surface [10], which means that the corrosion process needs to be regulated in order to achieve the ideal amount of released zinc ions so as to not cause any adverse effect on the organism. The problem can be solved by the passivation of the material surface by oxides or proteins [10,11,12], which affect the ion release. The process of passivation by proteins normally leads to a change of the corrosion rate by mutual relation between proteins and individual materials [13]. In addition, it is well known that the oxide layer leads to a decrease in material corrosion rate [14]. The enhancement of cell adhesion could be achieved also by the addition of biocompatible substances, which simulate the environment of the human body (e.g., hydroxyapatite). The cells adhere preferentially on those places, and the presence of those substances decreases the surface of the exposed metal and the degradation rate as a consequence [15].

The hydroxyapatite (HA) is an inorganic compound abundantly occurring as a part of the human bones. Many research groups confirmed the positive effect of HA on the regeneration of tissues [16,17]. According to those findings, HA is often used as a part of permanent and biodegradable implants [18,19,20,21]. The hydroxyapatite is widely used for the preparation of scaffolds, layers and composite materials with a metallic matrix [22,23,24]. The advantages of HA are mainly connected with its bioactive behavior [25]. In the case of biodegradable materials, the inclusion of the HA seems to be a more suitable method than the creation of the HA layer. HA is not degradable under physiological conditions; therefore, its surface layer would hinder the corrosion of the zinc substrate and the implant would not be degradable as a consequence.

The extrusion is a process which allows the preparation of materials with minimum defects in the structure. The advantage of this manner of compacting is evident, especially in the case of a composite material consisting of two compounds with a significant difference in melting temperatures. The extrusion leads to the creation of high-quality connections between the particles of the material with the lower melting temperature and enhances the mechanical properties of the composite material [26,27,28].

This study is focused on the characterization of a zinc-hydroxyapatite composite (Zn/HA 8 wt.%) prepared for the first time by the extrusion. Pure zinc prepared under the same conditions as the composite, was used as reference material. Based on our previous study [29], the amount of the hydroxyapatite (8 wt.%) reinforcement was chosen in order to determine the influence of processing on the material properties. The study extends the current knowledge about the behavior of zinc/hydroxyapatite composites and their potential usability in the human organism.

## 2. Materials and Methods

**Figure 1:**
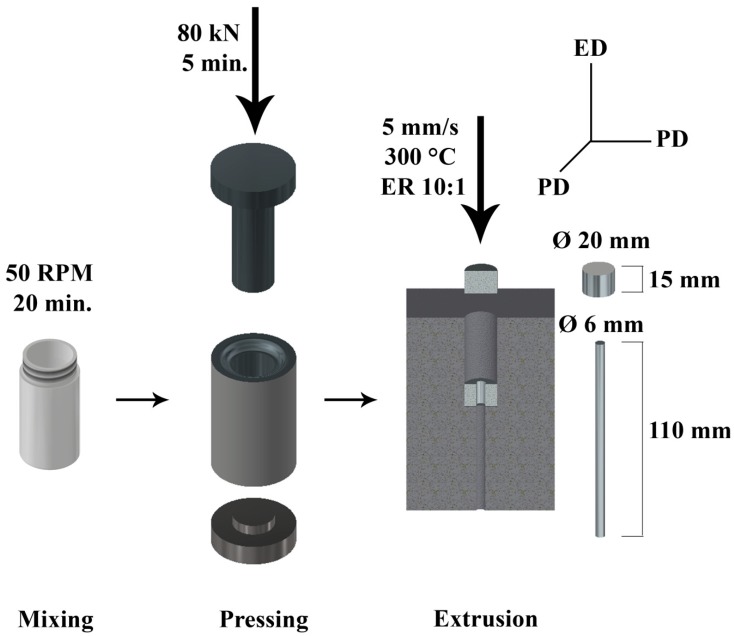
Schema of the Zn/HA8 composite preparation with the processing conditions of individual steps and with the description of the sample directions (ED—extrusion direction, PD—perpendicular direction).

Pure zinc (Zn; AlfaAesar, Ward Hill, MA, USA, purity 99.9%, 44–105 µm) and hydroxyapatite (HA; Medicoat, Etupes, France, 45–125 µm) powders were mixed for 20 min at 50 RPM in an air atmosphere using a Turbola T2C mixer (WAB-GROUP, Muttenz, Switzerland). The weight ratio of the powders in the mixture was 92 wt.% of Zn: 8 wt.% of HA. After the mixing, the powder mixture (Zn/HA8) and unmixed pure zinc (Zn) were pressed into tablets with 20 mm in diameter and 15 mm in height using a universal testing machine LabTest 5.250SP1-VM (LaborTech Inc., Opava, Czech Republic). Conditions used for the pressing process are shown in Figure 1. The billets of both materials were extruded into cylindrical rods with a diameter of 6 mm at a ram speed of 5 mm·s^−1^ and at an extrusion ratio (ER) of 10:1 and extrusion temperature of 300 °C. The extruded rods were cooled by water, immediately after the process, in order to prevent coarsening of grains by post-dynamic and static recrystallization. The ends of the samples (1 cm) were cut off in order to remove the parts of the material, which can, with high probability, possess some microstructural inhomogeneity. The process mentioned above is summarized in Figure 1.

The specimens intended for the microstructure investigation were ground using P180-P4000 sandpapers and polished using an Etosil E suspension (0.06 µm, pH ~7). The samples were etched using a chromium oxide solution composed of 20 g CrO_3_, 1.5 g Na_2_SO_4_ and 100 mL H_2_O. The microstructure of the prepared samples was analyzed using a light metallographic microscope Olympus PME3 (Olympus; Tokyo, Japan) and scanning electron microscope TescanVega3 LMU (SEM; TESCAN, Brno, Czech Republic) equipped with an energy dispersive spectrometer Oxford Inca 350 (EDX; AZtec, Oxford Instruments, Abingdon, UK). The amount of the hydroxyapatite, the porosity, and pore distribution were determined by image analysis from the micrographs and X-ray elemental maps of the polished sample surfaces using the ImageJ software (Version 1.42q, National Institute of Health, USA). The densities and porosities of the specimens were calculated according to Equations (1) and (2).

(1) $$\rho_{theor.}=\rho_{Zn}\cdot \;w_{Zn}+\rho_{HA}\cdot \;w_{HA}$$

(2) $$\varepsilon =\left(1-\frac{\rho_{real}}{\rho_{theor.}}\right)\times 100$$

where *ρ* (Zn = 7.14 g/cm^3^, HA = 3.08 g/cm^3^) is a density of individual components, *w* is the amount of components in weight percent, and *ε* is a theoretical porosity. The value *ρ(real.)* is a tabulated value of density for zinc, and the theoretical one is the density of composite calculated using Equation (1).

The microstructure of the prepared materials was also analyzed using a FEI 3D Quanta 3D field-emission-gun DualBeam microscope (SEM; Thermo Fisher Scientific, Waltham, MA, USA) equipped with an electron back scatter diffraction detector (EBSD) TSL/EDAX Hikari (Ametek, Berwyn, IL, USA). All the information like grain size, IPF (inverse pole figure) maps, and texture intensity were evaluated using EDAX OIM Analysis v8 software (Version 8.0, Amatek, Berwyn, IL, USA). The samples were orientated, in the chamber, perpendicularly to the extrusion direction. For the EBSD analysis, the samples were electrochemically polished in a H_3_PO_4_/ethanol (1:1) solution at 4V. The IPF maps were acquired from the sample surfaces with 500 × 800 µm dimension and the step 1 µm was used.

The testing of mechanical properties was done using the universal testing machine LabTest 5.250SP1-VM (LaborTech Inc., Opava, Czech Republic). Three samples were used for the compressive and three for the flexural testing. For the compressive testing (ASTM E9–19 Standard), cuboids with a height of 6 mm and a side length of 4 mm were used. For the flexural testing (ASTM E290–14 Standard), the samples with 20 mm in height and a square base with the side length of 4 mm were used. All the measurements of the mechanical properties were performed at laboratory temperature. The Vickers microhardness with a load of 1 kg (HV1) was measured using a micro-hardness tester Future-Tech FM-700 (Future-Tech Corp., Tokyo, Japan).

The corrosion behavior of the specimens was tested by immersion and electrochemical measurements. The cylindrical samples with both a diameter and a length of 5 mm were ground using P2500 SiC sandpaper. Before the exposition, the samples were ultrasonically cleaned in ethanol and acetone to prevent the impact of surface impurities on the testing. The immersion tests were performed in simulated body fluid (SBF27) [30] at 37 °C, and the time of exposition was 336 h (14 days). The pH development (3 samples) was controlled every second day of the exposition due to the characterization of solution behavior in time. The ratio between sample surfaces and the volume of SBF27 was 80 mL/cm^2^. The Zn^2+^ ion release was analyzed from the solution by an AGILENT 280 FS AA SPECTROMETER (AAS; Agilent Technologies, Mulgrave, Australia). The corrosion products, formed on the samples, were analyzed by the SEM-EDX and PANalytical X’Pert PRO powder diffractometer with a Co anode (λ = 0.1789 nm) in a Bragg–Brentano geometry (XRD; PANanalytical, Almelo, Holland). Subsequently, the corrosion products were removed by a chromium oxide solution and the degradation rate was evaluated from the weight losses (Equation (3)), where Δ*m* is the difference in sample weight before exposition and after chemical removal of corrosion products, *S* is the sample area, *ρ* is the density of the material and *t* is time (ASTM G31-72).

(3) $$v_{kor}=\frac{\Delta m\cdot t}{S\cdot \rho }$$

The polarization resistance and cyclic polarization measurements were performed using a potentiostat Gamry Reference600 (Gamry, Warminster, PA, USA). The conditions of electrochemical measurements are summarized in Table 1. The measurements were performed in the SBF27 solution at 37 °C using a standard three-electrode setup, where the Ag/AgCl (SSCE) and a graphite electrode were used as the reference and the counter electrode respectively. The corrosion rates of the Zn/HA composite and pure zinc were evaluated from the polarization resistance by linear approximation of the curves between −10 and +10 mV using an Echem Analyst software. The polarization resistance was measured twice, and the individual measurements were separated by 11 h of potential stabilization. After the polarization resistance measurements, the cyclic polarization was measured at the conditions mentioned in Table 1.

## 3. Results and Discussion

### 3.1. Microstructure

**Figure 2:**
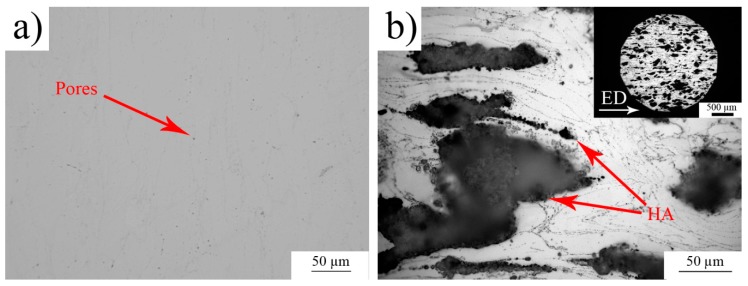
Micrographs of (**a**) pure zinc and (**b**) Zn/HA8 composite structures.

The zinc and hydroxyapatite initial powders were characterized in our previous work [29]. The porosities of the extruded samples were calculated according to Equations (1) and (2) and the resulting values were 10% for the composite and 1.1% for the pure zinc (Table 2). The value of the porosity of the Zn matrix also includes the pores between individual HA particles, which formed a major part of the porosity in the Zn/HA8 composite (Figure 2b). It means that the connection between individual HA particles was the weakest point of the composite. That was due to a low temperature during the extrusion process. To achieve strong diffusion connections between the HA particles, temperatures higher than the melting point of zinc (420 °C) would be necessary. In addition, the HA is not reacting with zinc. All that information suggests that only mechanical connections between Zn and HA were formed. In Table 2, the porosity of HA describes only the porosity of individual HA particles (~15%) recalculated to the whole volume of the sample. The porosity and its connectivity seem to be the most important factor for this type of composite materials. The character of porosity significantly affects all the characteristics (structural, mechanical, corrosion) of the composites. The value of 10% of porosity is in the literature mentioned as a limit which defines the connectivity type of a porous system. Higher values (>10%) often characterize the interconnected net of pores through the material [31]. We tried to evaluate the porosity value in the extruded samples also by theoretical calculations (Equation (2)) because of the high importance of this parameter. All the results suggested that the system of pores was closed in both extruded materials. It can be seen in Table 2 that the density of the extruded zinc was close to the tabulated value for bulk zinc (7.14 g/cm^3^). This means that the extrusion process eliminated almost all defects in the structure of the material. On the contrary, the density of the composite was lower by approximately 5.5% compared to its theoretical density calculated according to Equation (1) (6.45 g/cm^3^). It was most likely caused by the porosity, which was confirmed by optical observations.

The differences between the theoretical and real value of the densities were caused by the occurrence of the pores (visible in Figure 2). In the case of Zn/HA8 composite, the HA particles were partially broken during the extrusion process and the fragments formed lines parallel with the extrusion direction (Figure 2b). It can be seen in Figure 2b that the distribution of HA particles was homogeneous across the sample. The porosity between Zn–HA particles was negligible due to the plastic flow of zinc around the HA particle during the extrusion (Figure 2b). In addition, the metallographic examinations confirmed that the pore system was closed in both materials. That is crucial from the view of mechanical and corrosion properties.

**Figure 3:**
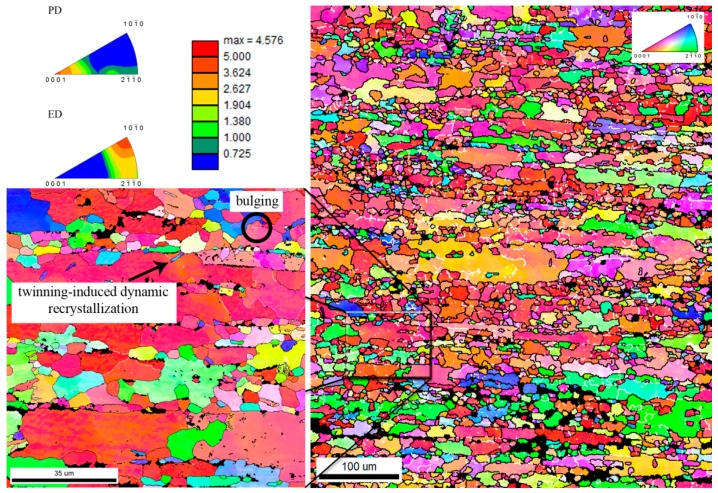
Inverse pole figures (IPF) and IPF maps (perpendicular direction (PD)) of pure zinc with characteristic signs of dynamic recrystallization.

**Figure 4:**
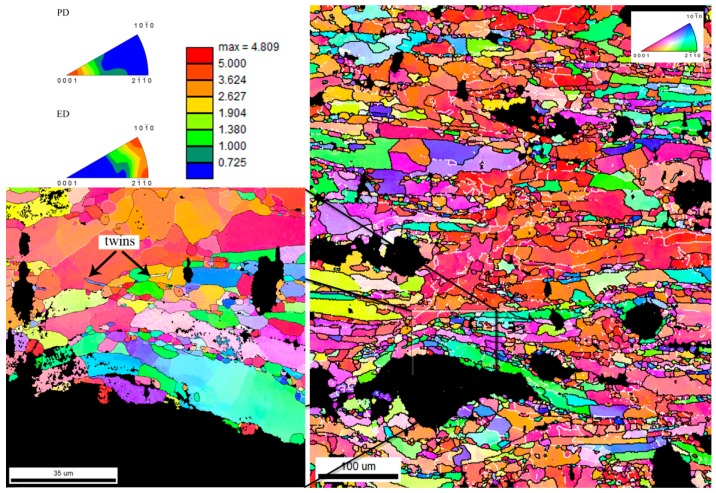
Inverse pole figures and IPF maps (PD) of Zn/HA8 with visible twins in the structure.

The specimens were also analyzed by EBSD in order to determine the influence of the HA particles on the recrystallization process. From the IPF maps of pure zinc (Figure 3) and Zn/HA8 (Figure 4), it is evident that the dynamic recrystallization (DRX) took place during the extrusion. The signs of the dynamic recrystallization process were bulging, chain-like structures and the formation of new grains in the inner structure of twins (twinning-induced dynamic recrystallization) [32]. Generally, the temperatures above 0.5 T_m_ often lead to discontinuous recrystallization and the higher stresses lead to continuous recrystallization. Based on that, it can be assumed that at a temperature of 300 °C (0.75 T_m_ of Zn) the primary process was discontinuous dynamic recrystallization (dDRX). This statement was confirmed for both materials and is shown in Figure 3 and Figure 4. Bulging and only low number of the low angle boundaries pointed also to the domination of the dDRX in both materials. It was evident from the IPF maps that the recrystallization process occurred in the Zn/HA8 composite in two different types of locations. The first types of locations were the grain boundaries of the original (parent) grains, and the second type of locations were the Zn-HA interfaces. It suggests that the interfaces between the HA particles and the Zn matrix acted as nucleation centers. However, the process of nucleation was most likely limited only to some areas in which a critical strain was achieved. Due to that, the recrystallized grains were not observed around all HA particles. The grain size of pure zinc and Zn/HA8 composites were determined from the EBSD data and the results shown relatively comparable values for both materials. The grain size was characterized by a bimodal distribution, which is obvious from the IPF maps. It was caused by the non-recrystallized grains in the structure (20–40 µm) and by recrystallized grains in the mentioned locations (up to 10 µm). The inverse pole figures (IPF) confirmed the common texture of hexagonal metals (with c/a ratio ≥1.63) after the extrusion, where the planes {1010} are dominating in the extrusion direction [33]. The texture was slightly affected by the presence of HA particles which led to an increase of intensity in the extrusion direction compared to pure zinc. This was evident {2110} from the inverse pole figures shown in Figure 3 and Figure 4 and supported by the literature [34].

### 3.2. Mechanical Properties

**Figure 5:**
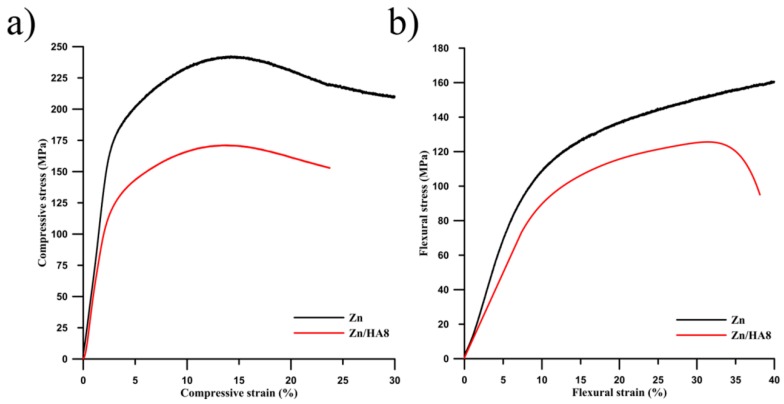
Engineering (**a**) compressive and (**b**) flexural curves of the extruded Zn and Zn/HA8 composite.

Engineering compressive and flexural curves are shown in Figure 5 and the values of the important mechanical characteristics obtained from these curves are listed in Table 3. It is obvious from those curves that the HA acted as a defect rather than a reinforcement. This can be attributed to the poor or even missing diffusion connections between the HA particles and the Zn matrix (see Section 3.1). Due to that, the mechanical characteristics were affected mainly by the quality of Zn connections and by the defects, especially by pores in the structure. The extrusion process significantly decreased the porosity and increased all the mechanical characteristic of the materials in comparison to the sintered samples containing the same amount of HA (Table 3) [29]. The compressive curves show that the compressive yield strength of the Zn/HA8 composite (113 MPa) was lower than that of the pure zinc (154 MPa) by approximately 30%. Equation (4) was used for the evaluation of the direct HA influence on the decrease of the composite compression yield strength.

(4) $$\sigma_{c}=\sigma_{Zn}\cdot V_{Zn}+\sigma_{HA}\cdot V_{HA}$$

where *σ_c_* (*σ_Zn_*, *σ_HA_*) represents the compressive yield strength of the Zn/HA8 composite (extruded Zn, HA) and *V* represents the volume fraction of individual components. Based on the available literature, the value of 10 MPa was used for the *σ_HA_* value [35]. The resulting value was 129 MPa and the slight difference between this value and the value obtained by our measurements (113 MPa) can be explained by the differences of the porosities (or other defects). The determination of the ultimate flexural strength of the extruded zinc was not possible due to the linear increase of the stress. It means that the number of defects initiating crack formation and the subsequent fracture was strongly limited in this material. As a consequence, the material showed a plastic behavior without the fracture of the sample. The Zn/HA8 composite showed a relatively high plastic region in the flexural curve (Figure 5b) even though the brittle HA particles occurred in its structure. It suggests that the HA particles were well embedded in the zinc matrix. Despite that, cracks formed in the material and fracture took place as a consequence. Similarly, as in compression, the flexure yield stress of the composite (76 MPa) was approximately about 30% lower than that of the pure Zn (106 MPa).

The mechanical properties of Zn/HA composites and pure zinc prepared by various methods are summarized in Table 3. The relationships between the content of HA, the preparation method and mechanical properties are obvious. The extrusion process leads to the preparation of materials possessing a high-quality diffusion connection between zinc particles and a limited number of defects (pores, etc.) occurring in the structure. As a result, relatively high values of mechanical properties have been observed for those materials. In contrast, only poor diffusion connections were formed in the case of the sintered samples [29] and the resulting mechanical performance was mainly influenced by the strength of those connections. Namely, the high amount of HA in the zinc structure increases the number of present defects and it leads to the deterioration of mechanical properties [36]. The comparison of the mechanical properties of those materials with human bone is quite problematic, because the mechanical properties of human bone are dependent on many parameters, such as the type of bone, gender or the age (in summary by the density of the bone) [37]. According to that, the comparison of the properties is only rough. However, it seems that the properties of the extruded samples almost reach the values of mechanical properties of the cortical bone. This means that there is a possibility to obtain a material used as a cortical bone replacement by a slight change of the extrusion conditions or by a decrease of the HA content in the composites.

**Figure 6:**
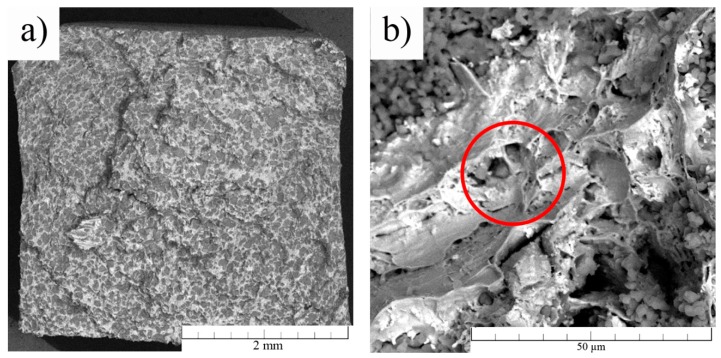
(**a**) Fracture surface of the extruded Zn/HA8 composite. (**b**) Detail of the fracture surface with signs of plastic deformation (circled in red).

Figure 6 shows the fracture surface of the extruded Zn/HA8 composite after the flexural testing. At first sight, the information from the fracture surface was in good agreement with the results of the flexural tests. The fracture surface of the Zn/HA8 composite contained visible areas of significant plastic deformation. This was connected with a high quality of the formed diffusion connections between the zinc particles. It is also clearly visible, that the fracture was spread through the material predominantly along the HA particles. This assumption was confirmed by the image analysis, where 64% of the area of the fracture surface was formed by the HA. This proves that the HA particles acted rather as the defects than reinforcements due to the weak or even none strength of the HA–HA and HA–Zn interfaces. In the case of extruded Zn, the fracture did not occur at all due to the tremendous plasticity of the material.

### 3.3. Corrosion Properties

**Figure 7:**
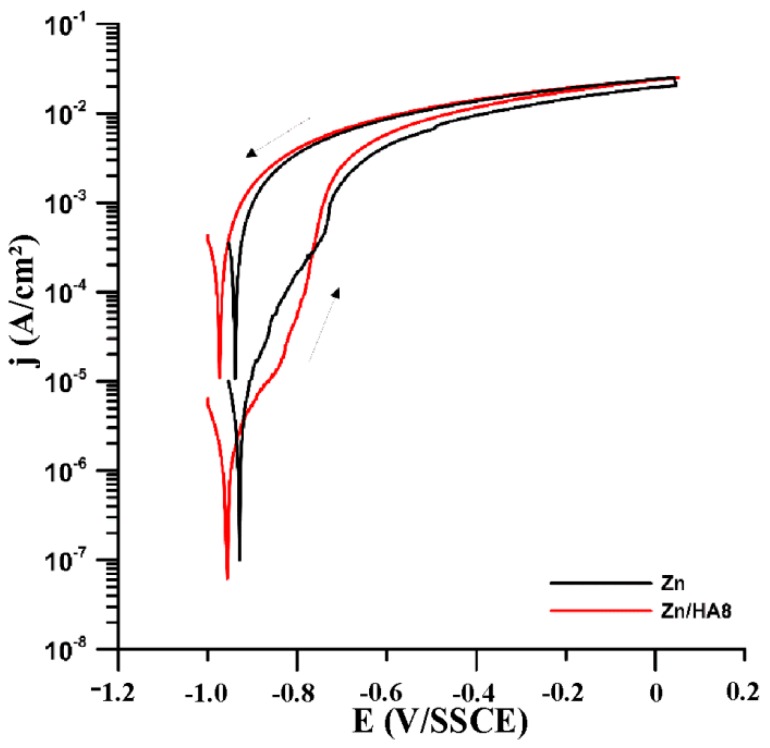
Polarization curves after 12 h of exposure in SBF.

In order to describe the corrosion behavior, measurements of polarization resistance, cyclic polarization and two-week immersion tests with subsequent analyses of corrosion products were performed. Table 4 contains the values of the polarization resistances that were measured after one and twelve hours of exposure. Individual measurements were separated by potential stabilization and after those measurements, the cyclic potentiodynamic polarization was measured (Figure 7).

It is apparent from the increase in polarization resistance (Table 4) that the surface of both materials was covered by a charge transfer limiting layer in the environment (SBF). The difference of values after one-hour of exposure is given by a different area of the materials, where the exposed area of corroding Zn in Zn/HA8 was lower due to the presence of the apparently electrochemically inactive hydroxyapatite. In the case of the Zn/HA8, the increase in resistance after 12 h of exposure was higher, which may be caused by faster growth of the layer. The difference was also evident from the values of the corrosion current density determined from the polarization curves measured at the end of the exposure. The existence of this layer was also confirmed by the shape of the curves in the anodic region close to the corrosion potential, where the anodic Tafel slopes have values of approximately 150–200 mV/decade. As the potential increased further, there was a significant current increase. This implies that the layer loses its protective character at higher potentials and zinc in both materials begins to corrode in the active state. It is also evident from the reverse polarization that there was no reformation of the protective layer during the measurement.

The corrosion rates of the Zn/HA8 composite and pure zinc, evaluated by immersion tests were 0.40 and 0.15 mm/a, respectively. The most interesting point was the increase of the Zn/HA8 degradation rate in comparison to pure zinc, during the immersion tests. It is well known that the HA did not participate in the corrosion process on the macroscopic scale. This means that the higher content of HA in the material should lead to a decrease in the exposed surface as well as in the degradation rate. A possible explanation of the opposite behavior is connected mainly with the exposition time. It means that with increased time, the corrosion medium penetrated into the material structure of the Zn/HA8 through the pores in the HA particles and the surface of exposed Zn was increased by that. The pore system in the zinc matrix was closed, which means that the changes caused by the corrosion process took place only in the “first” layer. The thickness of the “first layer” can be described as the height of the largest HA particle. Those assumptions were confirmed by the microscopic observations before and after the chemical removal of the corrosion products. Before the process, the HA particles were captured in the material by the corrosion products. On the contrary, the HA particles were released out of the composite structure after the process. The non-releasing of HA particles during material degradation is the crucial fact from the point of view of the material usability for biodegradable applications. The ion release after the immersion test was measured by atomic absorption spectroscopy, and it was found that the concentrations of released Zn^2+^ ions in the solution were 50 and 29 µg/cm^2^/day for the pure zinc and Zn/HA8 respectively. Those results show the possibility of using the materials for biodegradable applications without exceeding the recommended daily allowance (12.3 mg/day) [42], even with an implant of larger dimensions. It suggests that the zinc ions were bonded predominantly in the solid corrosion products, especially in the case of Zn/HA8 composite. It can be expected, based on the mentioned results, that the release of the HA particles after the pickling process had a major impact on the increase of the degradation rate of the Zn/HA8 composite obtained from the weight losses after the immersion test.

**Figure 8:**
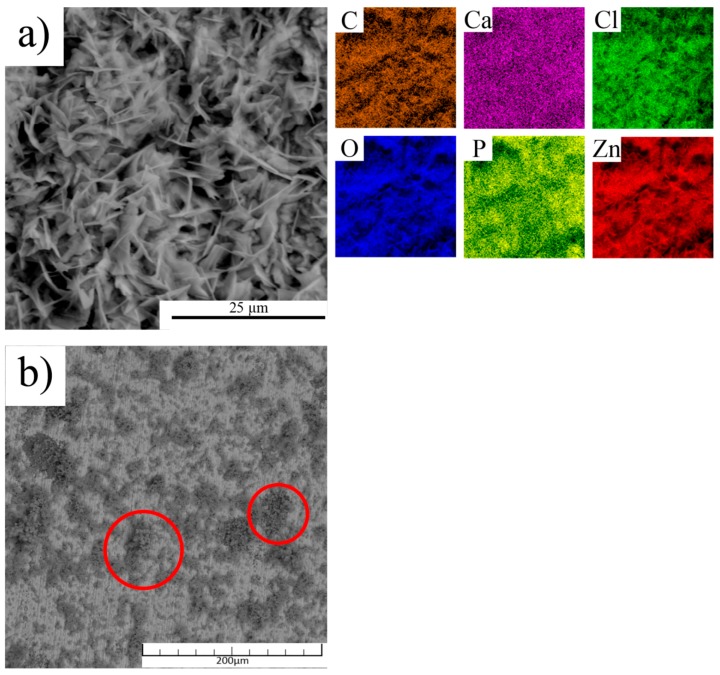
(**a**) The SEM image and X-ray elemental maps of corrosion products occurred predominantly on the upper layer and (**b**) SEM image showing the precipitation HA on the HA particles.

The corrosion products were characterized by EDS and XRD analyses. Elemental analysis revealed the presence of C, Ca, Cl, O, P and Zn. It was obvious that the phosphates, carbonates, and chlorides, as simple or complex substances, formed the corrosion products. Based on our previous study [43], the presence of simonkolleite Zn_5_(OH)_8_Cl_2_ (H_2_O) and hydrozincite Zn_5_(OH)_6_(CO_3_)_2_ can be expected. All those assumptions were confirmed by XRD analysis. However, the composition of the corrosion products was not the same over the entire surface of the samples. In addition, the preferential precipitation of the HA from the solution on the HA particles in the composite was observed (Figure 8b). Especially in the case of biodegradable metals, this is quite an important fact from the point of view of the tissue/implant interaction enhancement.

**Figure 9:**
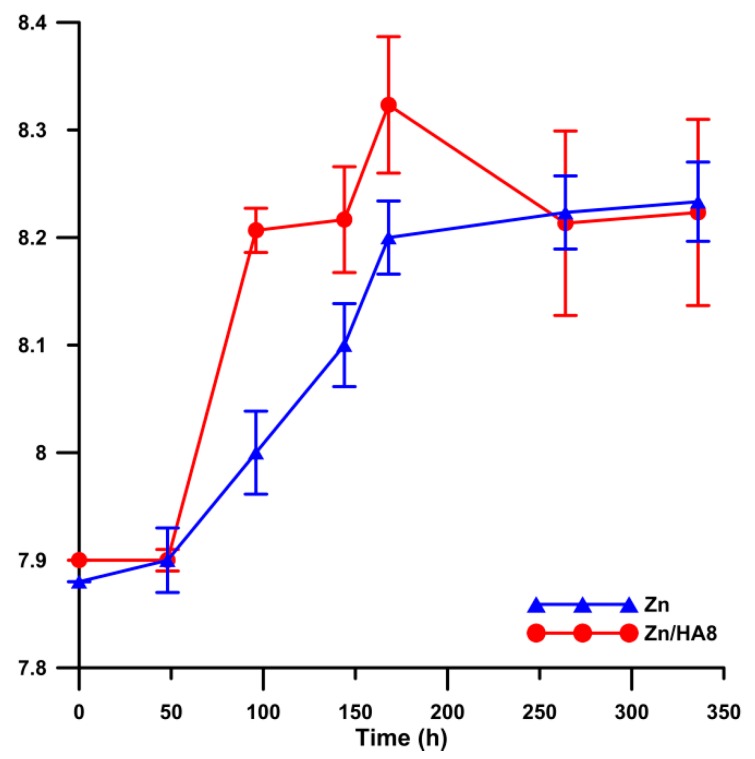
Development of pH during the immersion tests.

The determination of the corrosion behavior during the immersion tests included the measurement of pH during the exposure. It is obvious in Figure 9 that the pH was stabilized at the value of about 8.2 for both materials. It is known that the increase of pH in SBF stimulates the precipitation of hydroxyapatite from the SBF solution [44]. The precipitation of HA can significantly enhance the bone ingrowth and formation of the new tissue on the material/tissue interface [45]. In addition, the precipitation increases the amount of substances widely occurring in the human body and due to that, it decreases the possibility of a negative reaction of the organism to the material. Based on Figure 8b and Figure 9, it is possible that the pH locally increased around the HA particles and enhanced the precipitation of the HA from the solution. This increase could be caused by the penetration of the medium into the material structure, where the corrosion process was most likely accelerated by the presence of crevices on the Zn–HA interfaces. In addition, the acceleration led to a higher production of OH^−^ ions on the surface by the depolarization reaction and to a faster increase of pH compared with pure zinc. That assumption was particularly confirmed by Figure 8b, where it is visible that the HA did not precipitate on the zinc matrix of the composite. The deflection between 168 and 216 h could be caused by the coverage of HA particles with the HA from the solution and by the creation of Zn corrosion products on the Zn–HA interface. The formed compounds could create a barrier defending the solution flow in the material structure and led to a subsequent decrease of pH to the stable value.

## 4. Conclusions

In this study, the Zn/HA8 composite was successfully prepared, for the first time, by the extrusion process. The most important observations can be summarized in the following points:

- The extrusion process significantly enhances the plastic behavior of the material in comparison to other methods, which were studied previously.
- Recrystallization of the Zn matrix during the extrusion was enhanced in the surrounding of the HA particles.
- The HA particles act as defects in the structure from the point of view of the mechanical behavior.
- The presence of the HA particles decreases a mechanical performance (compressive and flexural) approximately by 30% in comparison to pure zinc prepared in the same way.
- The HA particles simplify the precipitation of HA from the solution.
- The corrosion rates of studied materials seem to be appropriate in relation to potential applications.
- All the mentioned results suggest the usability of those materials for specific biodegradable applications.

## Figures and Tables

**Table 1:** The conditions of individual electrochemical measurements.

Measurement	Stabilization (s)	Initial Potential (V)	Apex Potential (V)	Finish Potential (V)	Scan Speed (mV/s)
Polarization resistance	3600/39600	−0.02	−	0.02	0.125
Cyclic polarization	3600	−0.05	1	−0.05	2

**Table 2:** Calculated porosity and density of prepared samples.

Sample	Porosity (%)			Density (g/cm^3^)
	Zn Matrix/Zn	HA	Whole Sample	
Zn	1.1	−	1.1	7.060
Zn/HA8	8.5	1.5	10	6.094

**Table 3:** Overview of the mechanical properties of composites with different content of HA and pure zinc processed by various methods.

Samples	HV1	FYS (MPa)	UFS (MPa)	CYS (MPa)	UCS (MPa)	Ref.
Zn/HA8 Ex	44.7 ± 4.5	75.6 ± 8.2	127.6 ± 8.3	112.8 ± 5.1	168.9 ± 3.7	This study
Zn/HA8 SPS	34.3 ± 4.5	49.5 ± 4.4	63.6 ± 10.4	67.9 ± 7.4	88.9 ± 7.2	[29]
Zn/HA10 SPS	44.3 ± 2.6	−	−	45.2 ± 11.4	70.8 ± 6.3	[36]
Zn Ex	45.6 ± 2	106.4 ± 10.2	−	153.6 ± 11.1	243.8 ± 1.5	This study
Zn SPS	36.8 ± 1.4	78.8 ± 4.9	109.7 ± 9.88	92.1 ± 1.2	128.7 ± 1.7	[29]
Cortical bone	−	−	160–300	75–200	95–230	[38,39]
Cancellous bone	−	−	−	2–12	0.2–80	[40,41]

Note: SPS—spark plasma sintering, Ex—extrusion, Zn/HA8—zinc composite with 8 wt.% of HA, CYS—compressive yield strength, UCS—ultimate compressive strength, FYS—flexural yield strength, UFS—ultimate flexural strength.

**Table 4:** Polarization resistance and corrosion rates in simulated body fluid (SBF).

Samples	Rp after 1 h (kΩ·cm^2^)	approx. corr. Rate (µm/a)	Rp after 12 h (kΩ·cm^2^)	approx. corr. Rate (µm/a)
Zn	1.5	440.0	5.1	130.0
Zn/HA8	1.7	380.0	9.1	70.0
